# Diagnosis of Cutaneous Chromoblastomycosis and Its Response to Amphotericin B Therapy: A Case Report

**DOI:** 10.7759/cureus.28286

**Published:** 2022-08-23

**Authors:** Furqan Ul Haq, Hamza Yunus, Rafia Mukhtiar, Ammar Ahmad, Romesa Akram, Sumaira Imran

**Affiliations:** 1 Internal Medicine, Hayatabad Medical Complex Peshawar, Peshawar, PAK; 2 Obstetrics and Gynaecology, Hayatabad Medical Complex Peshawar, Peshawar, PAK

**Keywords:** amphotericin, copper pennies, muriform bodies, warty skin lesions, cutaneous mycosis, blastomycosis

## Abstract

Cutaneous chromoblastomycosis is a chronic subcutaneous fungal disease of the skin caused by *Blastomyces dermatitidis*, especially by *Fonsecaea*,* Phialophora*,and *Cladophialophora *species affecting the skin, lungs, intestines, stomach, and central nervous system. It is treated using itraconazole in mild cases and amphotericin B in severe cases. A six-year-old female child presented to the Dermatology Outpatient Department with pigmented brown to blackish tanned plaques and verrucous lesions on the face and extremities. These lesions were present for the past two and a half years and were slowly enlarging and involving other areas like the trunk. The lesions were proven on biopsy to be cutaneous blastomycosis. The patient was put on infusions of amphotericin B in a calculated pediatric dose. Her blood pressure and renal function tests were checked daily to avoid any electrolyte derangements, nephrotoxicity, and systemic infusion reactions caused by amphotericin B. Amphotericin B reduced the size of the cutaneous lesions, and treatment response was assessed on regular follow-ups. Chromoblastomycosis should be considered in the differential diagnosis to enable timely treatment and to prevent its lethal complications such as epidermoid carcinoma. Treatment should continue for two to three months until histopathology is negative to ensure complete eradication.

## Introduction

Chromoblastomycosis (CBM) is an insidious cutaneous granulomatous disease caused by dimorphic fungi such as *Blastomyces dermatitidis*, especially *Fonsecaea, Hialophora,* and *Cladophialophora* species. It primarily affects the skin, and rarely the central nervous system. Systemic spread occurs via the lymphatic system [1]. It is a non-pathogenic mold found in soil and decomposing organic matter which converts to a pathogenic form at body temperature [2]. Farmers and gardeners living in tropical areas are particularly susceptible to CBM because of the autoinoculation of these fungi into minor wounds. The disease progresses rapidly if not treated early with proper anti-fungal medications. It mainly affects the limbs and facial area. Patients often complain of pruritus and pain at the lesion site. Lesions are pigmented, brown-black, hyperkeratotic plaques and verrucous lesions. Muriform body (spherical to ovoid brownish bodies) forms due to the host immune response, while acanthosis and granuloma formation occurs because of chronic inflammation. Although extradermal involvement is rare, some studies have found pulmonary, gastrointestinal, and cerebral involvement, especially in immunocompromised patients [3,4]. The etiological species of CBM belong to the order Chaetothyriales and the family Herpotrichiellaceae*. Fonsecaea pedrosoi *and* Cladophialophora carrionii *are found in endemic areas [5].

These fungi are found in two forms: (1) saprophytic forms (conidia and hyphae), living on organic dead remains and decomposing them, and (2) muriform cells, pathogenic forms at body temperature. CBM has five types, namely, nodular, plaque, cicatricial, verrucous, and tumoral. Diagnosis is confirmed mainly on clinical examination of verrucous, hyperkeratotic papules, nodules, or plaques with a black dot appearance on their surface, which can be appreciated by the naked eye or on dermoscopy. In the early stages (isolated <5 cm lesion), CBM can be treated with cryotherapy/surgical excision, followed by itraconazole for six months, which usually results in a complete cure with negative repeat cultures and biopsies [6].

## Case presentation

**Figure 1:**
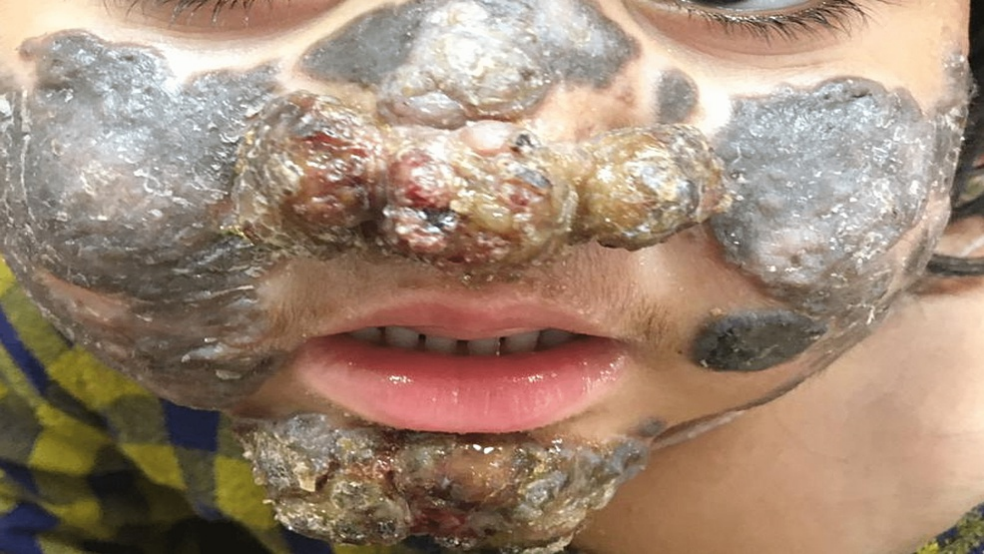
Chromoblastomycosis on the face and nose. Pigmented hyperkeratotic scaly plaques involving both cheeks and nasal bridge. Verrucous ulcerated lesions on the tip of the nose and chin with exudation of pus discharge.

**Figure 2:**
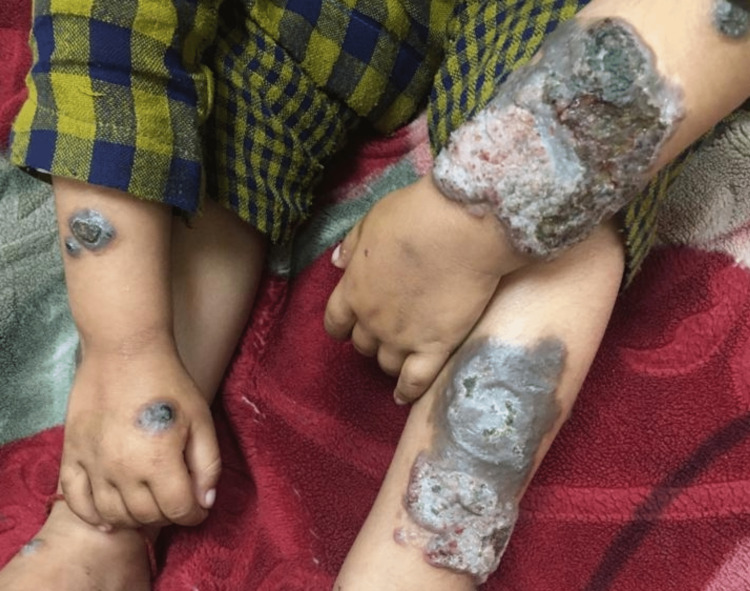
Chromoblastomycosis on the limbs. Pigmented hyperkeratotic scaly plaques involving both arms and legs.

A six-year-old girl presented to the Hayatabad Medical Complex Peshawar, Skin Outpatient Department with hyperkeratotic scaly itchy plaques and verrucous lesions on the face, including the nose, extremities, and the trunk but sparing the palms and genitalia for two and a half years. The skin lesions initially developed on the nose and then spread to adjacent areas such as the neck and face. Various topical skin medications (steroids, antibiotics, and antifungals) were used to treat them. The lesions did not respond and gradually the size of skin lesions increased and involved multiple areas, including the whole face, extremities, and trunk. She had no comorbidities or any chronic disorders (autoimmune diseases) that could have made her prone to develop CBM. She was evaluated one year back in 2021. A biopsy done at that time revealed the diagnosis of CBM. She was put on home medications such as antifungal syrups but was non-compliant and had no follow-up visits for treatment response. She then presented to the Skin Outpatient Department with extensive cutaneous involvement (lesions on the face, abdomen, forearms, and lower limbs) (Figures 1, 2), and was subsequently admitted for amphotericin B chemo infusions because her disease was extensive. Hepatic and renal parameters were normal. There was also suspicion of secondary bacterial infection because of pus discharge from the lesions. Culture and sensitivity testing of the pus showed growth of the bacteria *Providencia*, which was sensitive to meropenem, tigecycline, and imipenem. For this infection, she was given intravenous infusions of meropenem three times a day for one week based on pediatric calculated doses.

A significant reduction in hyperkeratotic and verrucous lesions was observed in the affected area after four months of treatment. There were no significant side effects with the use of amphotericin B. The renal profile and hepatic profile were closely monitored along with vital monitoring before and after amphotericin B infusions (5 mg thrice a week). On arrival, her baseline investigations were done (Table 1).

**Table 1:** Basic lab findings at initial presentation prior to the initiation of amphotericin B therapy. DLC: differential leukocyte count; RDW%: red cell distribution width; MCV: mean corpuscular volume; ESR: erythrocyte sedimentation rate; ALT: alanine transaminase; HIV: human immunodeficiency virus; HCV: hepatitis C virus; HBsAg: hepatitis B surface antigen; ICT: immunochromatographic test

Serial number	Clinical entity		Result	Normal range
1	Total leukocyte count		6.61 × 10³/µL	4–11 × 10³/µL
2	DLC	Neutrophils	41.2%	40–75 %
		Lymphocytes	40.8%	20–45 %
		Monocytes	10.2%	2–10 %
		Eosinophils	7.83%	0–6 %
3	Red blood cell count		5.01 × 10^6^/µL	4–6 × 10^6^/µL
4	Hemoglobin		11.5 g/dL	11.5–17.5 g/dL
5	MCV		75.1 fL	76–96 fL
6	RDW%		18.3%	11.5–14.5%
7	Platelet count		470 × 10³/µL	150 – 450 × 10³/µL
8	ESR		22	0–20 mm/1^st ^hour
9	Sodium		135 mmol/L	135–150 mmol/L
10	Potassium		4.34 mmol/L	3.5–5.1 mmol/L
11	Chloride		103 mmol/L	96–112 mmol/L
12	Blood urea		21 mg/dL	18–45 mg/dL
13	Creatinine		0.3 mg/dL	0.3–0.9 mg/dL
14	Total bilirubin		0.2 mg/dL	0.1–1 mg/dL
15	Alkaline phosphatase		88 U/L	<269 U/L
16	ALT		25U/L	10–50 U/L
17	Viral profile	Anti-HIV (ICT)	Negative	Negative
		HbsAg (ICT)	Negative	Negative
		Anti-HCV (ICT)	Negative	Negative

Histopathology reports

**Figure 3:**
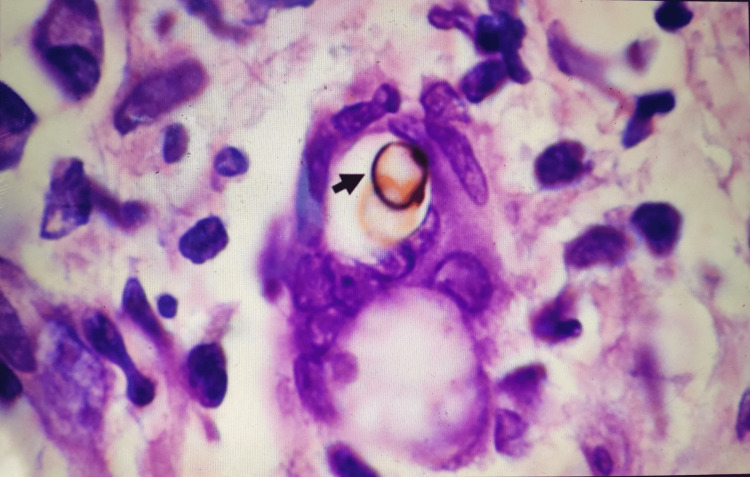
Mononuclear cell infiltrates with dark-brown, ovoid, copper penny-shaped sclerotic body shown by a black arrow. Reprinted by permission from the American Journal of Tropical Medicine and Hygiene. McDaniel P, Walsh DS. Chromoblastomycosis in Western Thailand. Am J Trop Med Hyg. 2010;83:448. doi: 10.4269/ajtmh.2010.10-0210 [7].

Biopsy showed a cellular infiltrate with dark-brown, ovoid, copper penny-shaped sclerotic bodies surrounded by mononuclear cells, histiocytes, and giant cells giving the appearance of a granuloma (Figure 3).

Treatment

Amphotericin treatment administered at the hospital is described in Table 2.

**Table 2:** Dose of Amphotericin B administered along with lab monitoring after each dose administered.

Date	Amphotericin dose	Creatinine	Alanine transaminase	Serum potassium	Serum sodium
8-2-2022	2 mg TD + 5 mg LD	0.3 mg/dL	20 U/L	4.1 mmol/L	133 mmol/L
9-2-2022	5 mg	0.3 mg/dL	22 U/L	4.5 mmol/L	135 mmol/L
15-2-2022	5 mg	0.2 mg/dL	21 U/L	4.3mmol/L	134 mmol/L
17-2-2022	5 mg	0.2 mg/dL	23 U/L	4.4 mmol/L	133 mmol/L

Vital signs were also assessed pre and post-amphotericin B administration to exclude any tachyarrhythmia or systemic reaction because of electrolyte imbalance. The patient tolerated the treatment well, as evidenced by normal labs such as renal function tests.

## Discussion

CBM is a slowly progressing indolent subcutaneous fungal infection that is often neglected, underdiagnosed, and untreated at an early stage because of its slowly evolving lesions which can be confused with similar infectious (lupus vulgaris, tertiary syphilis, leprosy, sporotrichosis, coccidioidomycosis, leishmaniasis, filariasis) and non-infectious (mycosis fungoides, psoriasis, sarcoidosis, squamous cell carcinoma) disease processes [8]. Patients usually live in tropical/subtropical areas where the environment is humid and favors the growth of the causative fungal organisms in soil [9]. Patients might provide a history of physical trauma to their exposed extremities and face likely leading to the inoculation of fungi into their wound. Some patients have occupations that involve the handling of plants and soil such as gardening and farming that predispose them to injury and subsequent disease development. On clinical examination, one can appreciate verrucous or hyperkeratotic papules, nodules, or plaques with a black dot appearance on their surface which can be appreciated by the naked eye or on dermoscopy. Definitive diagnosis of this disease relies on KOH skin prep or skin biopsy which reveals characteristic muriform cells (thick-walled, brown spherical-to-ovoid-shaped bodies, also known as sclerotic bodies or copper pennies). Other findings on histopathology of biopsied specimens include hyperkeratosis, pseudoepitheliomatous hyperplasia, and granulomas with Langerhan giant cells [10]. Early diagnosis of this rare disease may lead to early treatment with low morbidity and complete resolution of the infection without relapse.

Treatment of the disease depends upon its severity. Mild disease with one localized lesion less than 5 cm in size can be treated with surgical excision/cryotherapy with oral itraconazole for a few months. It usually results in a complete cure (clinical, histologic, mycologic) in the mild form of the disease. However, moderate-to-severe disease is treated more aggressively and for a prolonged duration using multiple systemic antifungals and mechanical measures (cryotherapy, heat therapy, and light-based therapy). Advanced forms of the disease show multiple relapses when the treatment is discontinued, and, sometimes, they are refractory to treatment. First-line antifungal in most cases is itraconazole 200-400 mg/day for 8-36 months, with cure rates ranging from 15% to 80%. Itraconazole can be hepatotoxic and has multiple drug interactions. Hence, liver function tests might be needed if used for a longer duration. The second most common antifungal is terbinafine 250-500 mg/day for several months to years depending upon disease severity [11]. Other antifungals that can be used include voriconazole, flucytosine, ketoconazole, and amphotericin B. Amphotericin has dose-limiting side effects, such as infusion reactions, electrolyte disturbances, and nephrotoxicity, which does not make it suitable for long-term use. Deng et al. found synergistic effects of amphotericin B and terbinafine against the majority of melanized fungi associated with CBM [12]. This combination could be an option for refractory cases of severe CBM not responding to first-line modalities.

Complications such as secondary bacterial infections causing osteomyelitis, tissue fibrosis causing stasis of lymph leading to lymphedema/elephantiasis, and, rarely, malignant squamous cell carcinoma can occur as the disease progresses.

## Conclusions

It is important to diagnose CBM early in its disease course with skin biopsy or KOH skin preparation looking for sclerotic bodies. Depending upon disease severity, treatment should be started promptly with patient education regarding the curability of the disease if compliance is achieved. Regular patient follow-up should be done to evaluate patients for any medication adverse effects and skin biopsy with cultures/KOH skin prep to ensure complete eradication of infection. Treatment should be continued for two to three months beyond the first negative skin biopsy to ensure complete cure. It is one of the neglected tropical diseases that has a chronic progressive course if not managed early. This case report highlights the clinical importance of early diagnosis of CBM with prompt treatment initiation and proper follow-up to eradicate the disease. Furthermore, clinical trials and meta-analyses are required to evaluate the efficacy of various treatment modalities in refractory CBM.

## References

[1] Albarillo FS, Varma GT, MacLeod SP (2018). Mandibular blastomycosis: a case report and review of the literature. Germs.

[2] Motswaledi HM, Monyemangene FM, Maloba BR, Nemutavhanani DL (2012). Blastomycosis: a case report and review of the literature. Int J Dermatol.

[3] Camara-Lemarroy CR, Soto-Garcia AJ, Preciado-Yepez CI, Moreno-Hoyos F, Hernandez-Rodriguez PA, Galarza-Delgado DA (2013). Case of chromoblastomycosis with pulmonary involvement. J Dermatol.

[4] Vyas MC, Joshi YR, Bhargava N, Joshi KR, Tanwar RK (2000). Cerebral chromoblastomicosis--a rare case report of cerebral abscess and brief review of literature--a case report. Indian J Pathol Microbiol.

[5] Queiroz-Telles F, Esterre P, Perez-Blanco M, Vitale RG, Salgado CG, Bonifaz A (2009). Chromoblastomycosis: an overview of clinical manifestations, diagnosis and treatment. Med Mycol.

[6] Brito AC, Bittencourt MJ (2018). Chromoblastomycosis: an etiological, epidemiological, clinical, diagnostic, and treatment update. An Bras Dermatol.

[7] McDaniel P, Walsh DS (2010). Chromoblastomycosis in Western Thailand. Am J Trop Med Hyg.

[8] Krzyściak PM, Pindycka-Piaszczyńska M, Piaszczyński M (2014). Chromoblastomycosis. Postepy Dermatol Alergol.

[9] Purim KS, Peretti MC, Fillus J Neto, Olandoski M (2017). Chromoblastomycosis: tissue modifications during itraconazole treatment. An Bras Dermatol.

[10] Queiroz-Telles F, de Hoog S, Santos DW (2017). Chromoblastomycosis. Clin Microbiol Rev.

[11] Kurien G, Sugumar K, Chandran V (2022). Chromoblastomycosis. https://www.ncbi.nlm.nih.gov/books/NBK470253/.

[12] Deng S, Lei W, de Hoog GS (2018). Combination of amphotericin B and terbinafine against melanized fungi associated with chromoblastomycosis. Antimicrob Agents Chemother.

